# Molecular characteristics of *eae*-positive clinical Shiga toxin-producing *Escherichia coli* in Sweden

**DOI:** 10.1080/22221751.2020.1850182

**Published:** 2020-12-10

**Authors:** Ying Hua, Xiangning Bai, Ji Zhang, Cecilia Jernberg, Milan Chromek, Sverker Hansson, Anne Frykman, Xi Yang, Yanwen Xiong, Chengsong Wan, Andreas Matussek

**Affiliations:** a Department of Microbiology, School of Public Health, Southern Medical University, Guangzhou, People’s Republic of China; b Division of Clinical Microbiology, Department of Laboratory Medicine, Karolinska Institutet, Huddinge, Sweden; c Division of Infectious Diseases, Department of Medicine Huddinge, Karolinska Institutet, Huddinge, Sweden; d State Key Laboratory of Infectious Disease Prevention and Control, National Institute for Communicable Disease Control and Prevention, Chinese Center for Disease Control and Prevention, Beijing, People’s Republic of China; e mEpiLab, School of Veterinary Science, Massey University, Palmerston North, New Zealand; f The Public Health Agency of Sweden, Solna, Sweden; g Division of Pediatrics, Department of Clinical Science, Intervention and Technology, Karolinska Institutet and Karolinska University Hospital, Stockholm, Sweden; h Department of Pediatrics, Queen Silvia Children's Hospital, Sahlgrenska University Hospital, Gothenburg, Sweden; i Department of Pediatrics, Institute of Clinical Sciences, Sahlgrenska Academy, University of Gothenburg, Gothenburg, Sweden; j Laboratory Medicine, Jönköping Region County, Jönköping, Sweden; k Department of Clinical and Experimental Medicine, Linköping University, Linköping, Sweden; l Division of Laboratory Medicine, Oslo University Hospital, Oslo, Norway; m Division of Laboratory Medicine, Institute of Clinical Medicine, University of Oslo, Oslo, Norway

**Keywords:** Shiga toxin-producing *Escherichia coli*, intimin, *eae* gene, gene diversity, hemolytic uremic syndrome, clinical significance

## Abstract

Shiga toxin (Stx)-producing *Escherichia coli* (STEC) can cause a wide range of symptoms from asymptomatic carriage, mild diarrhea to bloody diarrhea (BD) and hemolytic uremic syndrome (HUS). Intimin, encoded by the *eae* gene, also plays a critical role in STEC pathogenesis. Herein, we investigated the prevalence and genetic diversity of *eae* among clinical STEC isolates from patients with diarrhea, BD, HUS as well as from asymptomatic STEC-positive individuals in Sweden with whole-genome sequencing. We found that 173 out of 239 (72.4%) of clinical STEC strains were *eae* positive. Six *eae* subtypes (*ϵ*1, *γ*1, *β*3, *θ*, *ζ* and *ρ*) were identified *eae* and its subtype *γ*1 were significantly overrepresented in O157:H7 strains isolated from BD and HUS patients. *ϵ*1 was associated with O121:H19 and O103:H2 strains, and *β*3 to O26:H11 strains. The combination of *eae* subtype *γ*1 and *stx* subtype (*stx*_2_ or *stx*_1_+*stx*_2_) is more likely to cause severe disease, suggesting the possibility of using *eae* genotypes in risk assessment of STEC infection. In summary, this study demonstrated a high prevalence of *eae* in clinical STEC strains and considerable genetic diversity of *eae* in STEC strains in Sweden from 1994 through 2018, and revealed association between *eae* subtypes and disease severity.

## Introduction

Shiga toxin (Stx)-producing *Escherichia coli* (STEC), is an enteric foodborne pathogen that can be asymptomatic or cause mild diarrhea, bloody diarrhea (BD) or even hemolytic uremic syndrome (HUS) in infected humans [1,2]. HUS is the leading cause of acute renal failure in children with high morbidity and mortality [3]. Serotype O157:H7, associated with HUS and severe clinical outcomes, is the most predominant and virulent serotype among more than 400 serotypes that have been identified [2,4]. Nevertheless, since the early 2010s, non-O157 pathogenic serogroups, such as O26, O103 and O104, have been widely reported from HUS patients [5–7]. Ruminants, especially cattle, are the most important reservoir of STEC [8]. Direct contact with animals and their environment, consumption of undercooked beef, unpasteurized milk, other animal-derived products, contaminated water and vegetables are the main sources of human infections [9].

Pathogenicity of STEC in human is largely dependent on Stx, which is considered as the most important virulence factor. Stx, which is encoded by *stx* genes located on lambdoid prophages, has two types, Stx1 and Stx2, where Stx2 shows much stronger correlation with severe symptoms [10,11]. The duration of *stx* shedding is a main cause of secondary person-to-person (fecal-oral) transmission, the longer duration poses a high transmission risk [6]. Besides Stx, intimate adherence of STEC to the intestinal epithelium is also an important process in the STEC pathogenesis, it can cause attaching and effacing (A/E) lesions, which is a hallmark of STEC pathogenesis [12,13]. Intimin, encoded by the *eae* gene located in the locus of enterocyte effacement (LEE) pathogenicity island [14], plays a determinant role in the formation of A/E lesions by inducing the effacement of microvilli and forming of actin pedestals [15,16]. Also, intimin cooperates with its translocated intimin receptor-Tir, to trigger host signalling events and actin nucleation, thus inducing lesion formation [17]. Moreover, STEC injects a series of effector proteins into host cells through a type III secretion system (T3SS), which is encoded by LEE pathogenicity island, to play its pathogenic role [18].

The full length of the *eae* gene is about 2,800 base pairs (bp). *eae* has several subtypes, owning to its heterogeneous 3’ regions, which encoded protein that has been identified to be the intimin cell-binding domain (Int280a) [19]. There are at least 19 groups of *eae* subtypes, i.e. α, *β*, *γ*, *ϵ*, ξ, z, η, *θ*, τ, ι, κ, λ, μ, ν, υ, ο, π, *ρ* and σ, that have been defined so far [20]. It has been suggested that intimin alleles are responsible for different host specificity and tissue tropism [21]. Roger *et al.* showed that *eae* subtype *γ*1 appeared to be the most frequent among O157:H7 and O145:H28/H25/H^-^ strains [22,23]. A previous study has investigated the genetic diversity of intimin in atypical Enteropathogenic *Escherichia coli* (EPEC), where intimin *β*1 was suggested to be the most frequent subtype among atypical EPEC strains from diarrheal patients [24]. However, the molecular characteristics of *eae*-positive STEC strains, especially clinical strains, have rarely been described. Moreover, the relationship between *eae* subtypes and clinical symptoms, as well as duration of *stx* shedding remains to be addressed.

The aim of this study is therefore to investigate the *eae* subtypes and polymorphisms among clinical STEC strains isolated from STEC-positive individuals present with varying symptoms in Sweden, and to assess the association of *eae* subtypes with disease severity.

## Materials and Methods

### Ethics statement

The study was approved by the regional ethics committees in Gothenburg (2015/335-15) and Stockholm (2020-02338), Sweden, respectively.

### Strain collection and clinical information

A total of 239 STEC strains were isolated from STEC-infected individuals from 1994 through 2018 in Sweden. Clinical data of STEC patients were collected through reviewing medical records as well as routine praxis used for the STEC surveillance performed in Sweden. The duration of bacterial shedding was determined as the time period from the first *stx*-PCR-positive sample to the first negative sample, and clinical symptoms were classified into HUS, bloody diarrhea (BD) and non-bloody stool (NBS) [25].

### Whole-genome sequencing, assembly and annotation

Bacterial DNA was extracted and whole genomes were sequenced by Illumina HiSeq X platform at SciLifeLab (Stockholm, Sweden) and Ion Torrent S5 XL platform (Thermo Fisher Scientific, Waltham, Massachusetts, US) at The Public Health Agency of Sweden as previously described [26]. The Illumina sequencing reads were *de novo* assembled with SKESA (version 2.3.0) [27]. The Ion Torrent sequencing reads were *de novo* assembled with SPAdes (version 3.12.0) in “careful mode” [28]. The genome assemblies were annotated with Prokka (version 1.14.6) [29]. The assemblies of all strains in this study were deposited in GenBank with accession numbers and metadata shown in Table S1.

### Serotyping and stx subtyping

Serotype was determined by comparing assemblies to the SerotypeFinder database (DTU, Denmark) (http://www.genomicepidemiology.org/) using BLAST+ v2.2.30 [30]. The *stx* subtypes were determined by ABRicae version 0.8.10 (https://github.com/tseemann/abricate). An in-house *stx* subtyping database was created with ABRicate by including representative nucleotide sequences of all identified *stx*_1_ and *stx*_2_ subtypes. The assemblies were then used to search against the *stx* subtyping database. Multilocus sequence typing (MLST) was conducted *in silico* using the on-line tool provided by the Warwick *E. coli* MLST scheme website (https://enterobase.warwick.ac.uk/species/ecoli/allele_st_search). Sequences types (STs) were determined based on the seven housekeeping allelic genes (*adk*, *fumC*, *gyrB*, *icdF*, *mdh*, *purA*, and *recA*) profile.

### *Eae* subtyping and polymorphism analysis

The complete sequences of the *eae* gene were extracted from the genome sequences according to the genome annotation, and then aligned with reference sequences of all described *eae* subtypes downloaded from GeneBank. The genetic distances of *eae* subtypes were computed using the Maximum Composite Likelihood method by MEGA 7.0 software, and a Neighbor-Joining tree was generated with 1,000 bootstrap resamplings. As earlier described [20], a 95% nucleotide sequence identity cut-off value was used to characterize an innovative *eae* subtype. *eae* genotypes (GTs) based on *eae* sequence polymorphism was used to determine the diversity within each *eae* subtype.

### Comparison of clinical *eae*-positive STEC strains with strains from other sources

To assess the relationship of *eae*-positive clinical STEC strains in this study and strains from other sources, the MLST allelic profiles of *eae*-positive strains isolated from animals, meat and humans reported in a recent survey [20] were used for comparison. A minimum spanning tree was generated with BioNumerics software version 7.6 (Applied Maths, Belgium).

### Statistical analyses

Fisher's exact test was used to analyze the association between *eae* subtypes and bacterial features or clinical outcomes, the statistical significance was determined by Statistica12 (StatSoft, Inc. Tibco), *p*-value <0.05 was considered statistically significant.

## Results

### Prevalence of *eae* in clinical STEC strains

Among 239 clinical STEC strains, *eae* was present in 173 (72.4%) strains, including 56 HUS-associated strains, and 117 non-HUS strains (44 from patients with BD and 73 from individuals with NBS). All 65 O157:H7 strains and 108 (62.1%) non-O157 strains carried *eae* (*p*<0.0001). *eae* was overrepresented in strains from children (73.08%, *p*=0.011). The presence of *eae* was significantly associated with BD, HUS, and O157:H7 (Table 1). However, no association was observed between the presence of *eae* and the duration of bacterial shedding.

**Table 1.** Prevalence of *eae* gene in 239 STEC strains isolated from STEC-positive individuals.^a^

* eae*	No. (%)		*p*-value	No. (%)		*p*-value	No. (%)		*p*-value	No. (%)		*p*-value
	HUS (*n*=60)	Non-HUS (*n*=179)		BD (*n*=51)	NBS (*n*=128)		O157:H7 (*n*=65)	Non-O157 (*n*=174)		Adult (*n*=80)	Child (*n*=104)	
Positive	56 (93.33)	117 (65.36)	<0.0001*	44 (86.27)	73 (57.03)	<0.001*	65 (100.00)	108 (62.07)	<0.0001*	44 (55.00)	76 (73.08)	0.011*
Negative	4 (6.67)	62 (34.64)		7 (13.73)	55 (42.97)		0 (0.00)	66 (37.93)		36 (45.00)	28 (26.92)	

HUS: hemolytic uremic syndrome; BD: bloody diarrhea; NBS: non-bloody stool.

^a^ The association was analyzed between *eae* gene and clinical symptoms (HUS and non-HUS; BD and NBS), serotypes (O157 and Non-O157), age groups (Adult: ≥10 years; Child: <10 years), duration of bacterial shedding (Long: >24 days; Short: ≤24 days), only differences showing statistical significance were shown.

* Statistically significant difference.

### Diversity and subtype of *eae* in correlation with clinical outcomes

**Figure 1.**
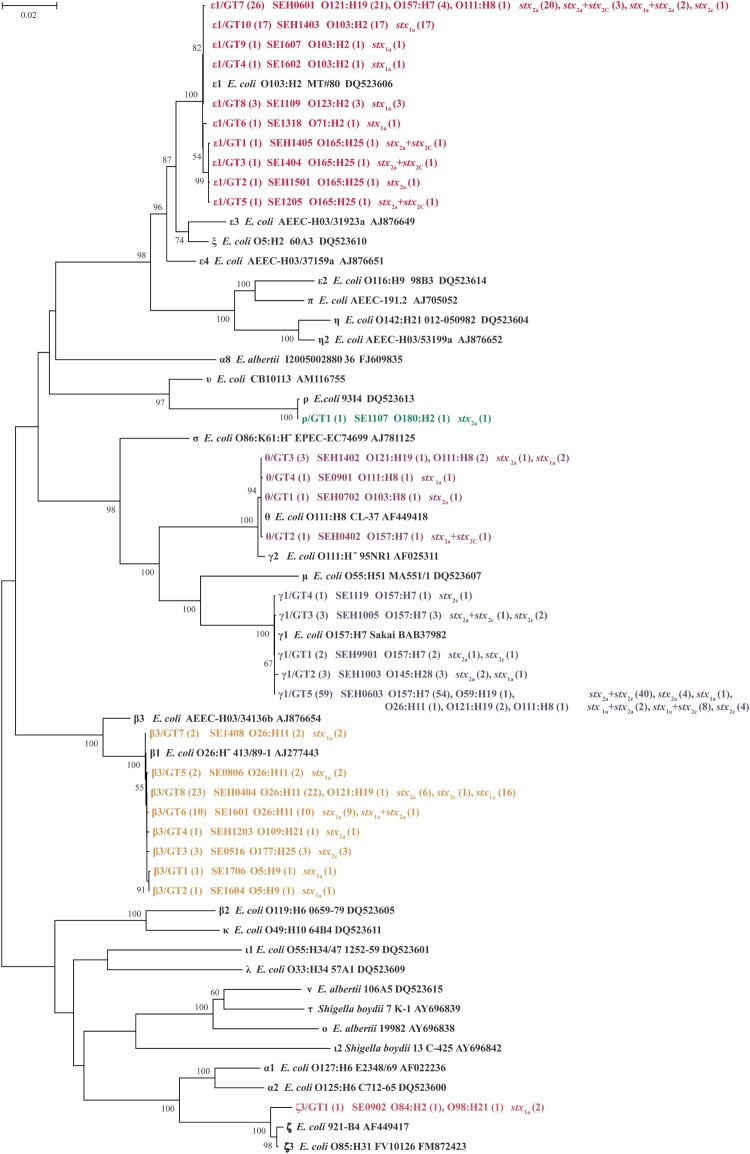
Phylogenetic relationships of 29 different *eae* sequences identified in this study and 30 *eae* subtypes reference sequences based on Neighbor-Joining method. The corresponding *eae* subtype (number of strains), strain name, serotype (number of strains), and *stx* subtype (number of strains) are shown. The *eae* subtypes/genotypes in this study are indicated in bold and different colors. Scale bar indicates genetic distance.

Six *eae* subtypes, namely epsilon1 (*ϵ*1), gamma1 (*γ*1), beta3 (*β*3), theta (*θ*), zeta3 (*ζ*3) and rho (*ρ*), were assigned in 173 *eae*-positive STEC strains. *γ*1 was present in 39.3% of all strains, being the most predominant subtype, followed by *ϵ*1 (30.6%) and *β*3 (24.9%). GTs were analyzed to determine the diversity within each *eae* subtype. Twenty-nine unique *eae* sequences were obtained, among which, *ϵ*1 subtype had ten GTs (GT1-GT10), followed by *β*3 (GT1-GT8), *γ*1 (GT1-GT5), *θ* (GT1-GT4), *ζ*3 (GT1) and *ρ* (GT1) (Figure 1). Isolates with the same *eae* subtype clustered together with the corresponding references (Figure 1). Notably, *γ*1 was statistically overrepresented in strains from BD and HUS patients, while *β*3 was related to non-HUS strains (Table 2). No association was found between *eae* subtypes and the duration of bacterial shedding or age of patients (Table S2).

**Table 2.** Association between *eae* subtypes and clinical symptoms or bacterial variables.^a^

eae subtype		Symptoms					Symptoms					Serotypes				
	No. strains	HUS (n=56)		Non-HUS (n=117)		*p*-value	BD (n=44)		NBS (n=73)		*p*-value	O157:H7 (n=65)		non-O157 (n=108)		*p*-value
		pos	prevalence	pos	prevalence		pos	prevalence	pos	prevalence		pos	prevalence	pos	prevalence	
ϵ1	53	14	25.00%	39	33.33%	0.27	13	29.55%	26	35.62%	0.5	4	6.15%	49	45.37%	<0.001*
γ1	68	33	58.93%	35	29.91%	<0.001*	19	43.18%	16	21.92%	0.01*	60	92.31%	8	7.41%	<0.001*
β3	43	5	8.93%	38	32.48%	<0.001*	11	25.00%	27	36.99%	0.18	0	0.00%	43	39.81%	<0.001*
θ	6	3	5.36%	3	2.56%	0.35	1	2.27%	2	2.74%	0.88	1	1.54%	5	4.63%	0.28
ζ3	2	1	1.79%	1	0.85%	0.59	0	0.00%	1	1.37%	0.44	0	0.00%	2	1.85%	0.27
ρ	1	0	0.00%	1	0.85%	0.49	0	0.00%	1	1.37%	0.44	0	0.00%	1	0.93%	0.44

HUS: hemolytic uremic syndrome; BD: bloody diarrhea; NBS: non-bloody stool; pos: number of positive strains.

^a^ The association was analyzed between *eae* subtype and clinical symptoms (HUS and non-HUS; BD and NBS), age groups, serotypes, as well as duration of bacterial shedding, only variables with differences showing statistical significance were shown in this table.

* Statistically significant difference.

### *Eae* subtypes and genotypes correlated with serotypes

In total, 173 *eae*-positive strains were typed into seventeen O serogroups and nine H types, which were assigned into 17 serotypes, O157:H7 (n=65, 37.57%) was the most predominant serotype. O157:H7 strains harbored *eae*-*γ*1 (60, 92.31%), *ϵ*1 (4, 6.15%) and *θ* (1, 1.54%). *γ*1 was found to be statistically associated with O157:H7, while *ϵ*1 and *β*3 were related to non-O157 serotypes (Table 2). Among non-O157 serotypes, we found that *ϵ*1 was associated with O121:H19 and O103:H2 serotypes, *β*3 was associated with O26:H11. The only two strains with *eae* subtype *ζ*3 were assigned to serotypes O84:H2 and O98:H21. Only O180:H2 strain carried *eae* subtype *ρ* (Table S3). All O121:H19 strains carried *eae* with one exception. *ϵ*1/GT7 was found to be linked to O121:H19 (*p*<0.0001).

### Association of *eae* and its subtypes with stx subtypes

Overall, six *stx* subtypes and combinations were identified in 173 *eae*-positive STEC strains, namely, *stx*_1a_, *stx*_2a_+*stx*_2c_, *stx*_2a_, *stx*_2c_, *stx*_1a_+*stx*_2a_, and *stx*_1a_+*stx*_2c_, among which *stx_2_* (57.2%) was more prevalent than *stx*_1_ (35.3%). We found that the presence of *stx*_1_+*eae* was significantly more prevalent in non-HUS STEC strains compared with HUS associated STEC strains. Notably, the presence of *stx*_2_+*eae* was significantly linked to HUS associated STEC strains, and *stx*_1_+*stx*_2_+*eae* was linked to BD-related strains (Table 3).

**Table 3.** Association between presence of stx + eae and clinical symptoms.

*stx* + *eae*	No. (%)		*p*-value	No. (%)		*p*-value
	HUS (*n *=* *56)	non-HUS (*n *=* *117)		BD (*n *=* *44)	NBS (*n *=* *73)	
*stx*_1_+*eae*	3 (5.36)	58 (49.57)	<0.0001*	14 (31.82)	44 (60.27)	0.003*
*stx*_2_+*eae*	51 (91.07)	49 (41.88)	<0.0001*	23 (52.27)	26 (35.62)	0.08
*stx*_1_+ *stx*_2_+*eae*	2 (3.57)	10 (8.55)	0.23	7 (15.91)	3 (4.11)	0.04*

* Statistically significant difference.

*stx*_1a_ was the most predominant *stx* subtype, with 35.3% of strains carrying this subtype. *stx*_1a_+ *stx*_2c_ only existed in strains carrying *eae*-*γ*1. *eae*-*ρ* subtype was only found in strains harboring *stx*_2a_. Notably, *ϵ*1 was associated with *stx*_2a_, *γ*1 was related to *stx*_2a_+*stx*_2c_, and *stx*_1a_+*stx*_2c_, *β*3 was linked to *stx*_1a_ (Table S4).

### Comparison of clinical *eae*-positive STEC strains with strains from other sources

**Figure 2.**
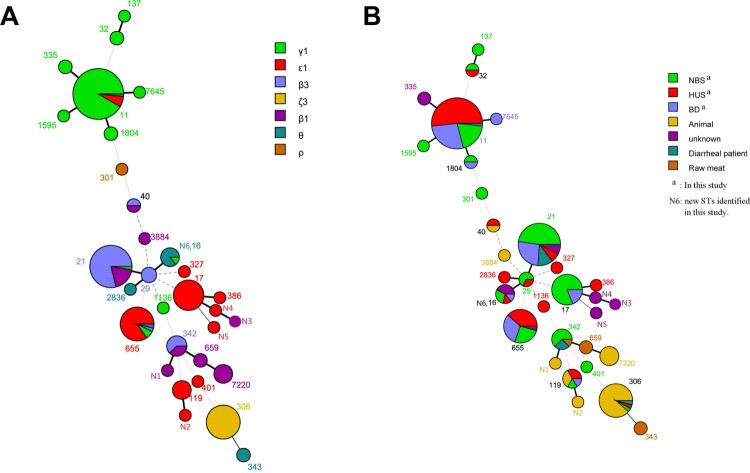
Minimum spanning tree of 22 STs from this study compared with 18 STs from other sources. Each circle represents a ST, with the pie divided proportionally to the number of isolates in that ST from different *eae* subtypes (A) or different sources (B).

Twenty-two sequence types (STs) were found in the 173 *eae*-positive STEC isolates (Table S1). ST11 was the most common sequence type, all 61 O157:H7 strains belonged to ST11. A minimum spanning tree was generated using 22 STs from this study and 18 STs from other sources reported previously [20]. Interestingly, isolates from the same source showed tendency to cluster closely. For instance, isolates from humans, independent on patients with BD, HUS, or individuals with NBS, clustered closely, while isolates from animals and raw meat showed closer relatedness. Notably, when grouped with *eae* subtypes, the majority strains belonging to the most predominant *eae* subtype *γ*1 were grouped closely with a few exceptions. Similarly, strains with the same other *eae* subtypes were more likely to cluster closely (Figure 2).

## Discussion

STEC strains harboring *eae* are suggested to be more pathogenic with a higher risk of developing HUS [31]. Little is known regarding the features and polymorphism of *eae* gene in STEC strains derived from patients as well as their association with disease severity. Here, we performed molecular characterization of *eae*-positive STEC strains from patients with a variety of symptoms as well as asymptomatic carriers. We found that 72.4% of clinical STEC strains were *eae* positive, out of which 37.6% were O157 strains, and 62.4% were non-O157 strains. All clinical O157 strains were *eae* positive, while 62.1% of non-O157 strains carried *eae*, which is much higher than that of reported in a recent study where only 9.5% of non-O157 strains carried *eae* [20]. We found that 99.3% of HUS associated STEC strains possessed *eae*, which was significantly higher than *eae* prevalence in non-HUS STEC strains (65.4%). Additionally, *eae* positive rate in strains isolated from patients with BD (86.3%) was higher than that of individuals with NBS (57.0%). It’s well-recognized that O157 is the primary cause of HUS [32–34]. We found that *eae* was significantly more prevalent in O157 strains, which may partially explain severe clinical outcomes of O157 strains.

The *eae* sequences in 173 STEC strains were classified into six subtypes, namely *ϵ*1, *γ*1, *β*3, *θ*, *ζ*3 and *ρ*. *eae*-*γ*1 and *ϵ*1 were the most common subtypes in this study. The prevalence of *eae* subtypes varies among studies. In a previous study, *β*1 and *ζ*3 were the most prevalent *eae* subtypes among STEC strains from different sources including diarrhea patients, raw beef and mutton, cattle, and yak [20]. *eae*-*γ*1 and *β*1 were reported to be the most widespread subtypes in STEC strains isolated from patients in Germany [35]. *eae* alleles examined in STEC strains isolated from ruminant animals also showed great genetic diversity. *β* and *ζ* were the most common *eae* subtypes in strains isolated from sheep, while *β* and *θ* were more prevalent in strains from cattle [36]. *ϵ*1 and *γ*1 were the most frequent *eae* subtypes among STEC strains isolated from healthy cattle [23]. The reason possibly lies in different sample sources and geographic distribution. It has been demonstrated that *eae* subtype *β*, *ϵ*, *γ*1, and *θ* are linked to more virulent strains [37]. Here, we found that *γ*1 was associated with severe clinical symptoms such as BD and HUS, highlighting the clinical significance of *eae* subtype *γ*1. However, the underlying mechanisms how different *eae* subtypes modulate the pathogenicity remains to be elucidated.

A diverse range of serotypes were observed among *eae*-positive STEC isolates. An earlier study showed association between serotypes and *eae* subtypes: O157 and O145 strains tended to harbor *γ*1, O103 and O121 harbored *ϵ*, O26 carried *β*, while O111 possessed *θ* and *β* [37]. In the present study, we observed a similar pattern. *γ*1 was significantly overrepresented in O157:H7 strains, also in line with a previous report of STEC strains derived from humans in Switzerland [22]. Similarly, *ϵ*1 was found to be prevalent in O121:H19 and O103:H2 strains. *β*3 was predominant in O26:H11 strains.

Notably, *ϵ*1 was found in four O157:H7 strains, which has been rarely reported before. In Germany, two O157:H16 strains isolated from diarrheal children carried *eae*-*ϵ* [38]. Another study reported O157:H16 strains isolated from water and meat also harbored *eae*-*ϵ* [39]. More data is needed to characterize the O157:H7 strains carrying *eae*-*ϵ* subtype.

The coexistence of *stx* and *eae*, especially *stx*_2_, are more likely to enhance virulence and increase the severity of clinical outcomes in humans than those carrying *stx*_1_ alone [40–42]. Consistently, we found that the presence of *stx*_2_+*eae* in STEC strains is strongly associated with HUS. Interestingly, the presence of *stx*_1_+*stx*_2_+*eae* was linked to BD, while *stx*_1_+*eae* was associated with NBS, supporting the evidence that the presence of *stx*_2_, rather than *stx*_1_, together with *eae* was associated with severe disease. Besides the finding that *eae* subtype *γ*1 was associated with HUS and O157:H7, *γ*1 was also found to be associated with *stx*_2a_+*stx*_2c_ and *stx*_1a_+*stx*_2c_, these are high virulent *stx* subtypes, which could also contribute to the severity of clinical symptoms.

Longer duration of *stx* shedding poses higher risk for the transmission of STEC strains from person to person, STEC-infected patients below 15 years old are usually associated with longer shedding duration [6,43]. Consistently, we found that children had a longer shedding duration than adults (unpublished data). Several genes were reported to be associated with prolonged duration of shedding [25]. In this study, we found that the presence of *eae* was associated with children. However, the presence of *eae* and subtypes has no association with the duration of *stx* shedding. As the information of age and the duration of shedding for some individuals is missing, further research is needed to understand the role of *eae* in children with longer duration of shedding.

In conclusion, here we describe the prevalence and genetic diversity of *eae* genes in clinical STEC isolates from Sweden from 1994 through 2018. Our results show that the majority of the clinical STEC isolates carry *eae* genes, which demonstrate highly genetic diversity. We found associations between *eae* subtypes and certain serotypes. Furthermore, *eae* subtype *γ*1 is associated with strains causing severe symptoms. However, no correlation was observed between the presence of *eae* gene/subtypes and duration of bacterial shedding. Our study proposes that the coexistence of *eae* subtype *γ*1 and *stx*_2_ or *stx*_1_+*stx*_2_, could be used as a risk predictors for severe symptoms of STEC infections.

## Supplementary Material

Table_S4.xlsx

Table_S3.xlsx

Table_S2.xlsx

Table_S1.xlsx
